# Giant Inguinoscrotal Hernia With Loss of Domain in a Patient With Alcoholic Liver Cirrhosis: A Case Report

**DOI:** 10.7759/cureus.87338

**Published:** 2025-07-05

**Authors:** Jesus A Olvera, Pedro Solis Marfil, Erick R Guerra Bocanegra, Carlo P Meza Hernandez, Sharon A Gomez Martinez

**Affiliations:** 1 General Surgery, Clinica Hospital ISSSTE Constitución, Monterrey, MEX; 2 General Surgery, Instituto Mexicano del Seguro Social, Monterrey, MEX; 3 General Surgery, Universidad del Valle de México, Monterrey, MEX; 4 General Surgery, Universidad Autónoma de Nuevo León, Monterrey, MEX

**Keywords:** abdominal wall, botulinum toxin, inguinoscrotal hernia, liver cirrhosis, progressive pneumoperitoneum

## Abstract

Inguinal hernias are commonly seen in clinical settings and may occasionally present in complex forms that pose significant surgical challenges. In its giant inguinoscrotal form with loss of domain, it presents a significant surgical challenge. These hernias are associated with considerable risks, particularly due to potential cardiorespiratory complications during reintegration of the intra-abdominal organs. The complexity increases in patients with comorbidities such as liver cirrhosis. We present the case of a 47-year-old male patient with alcoholic liver cirrhosis who developed a giant inguinoscrotal hernia containing loops of colon and small bowel. Treatment involved preoperative preparation with progressive pneumoperitoneum and botulinum toxin administration, followed by elective hernioplasty, which resulted in a favorable clinical outcome. This case underscores the importance of an individualized, multidisciplinary approach to optimize surgical outcomes in patients with complex medical conditions.

## Introduction

Inguinal hernia is the most common type of abdominal wall hernia, occurring predominantly in men with a male-to-female ratio of 4:1, and is more frequently seen during the productive years of life. Its main complications include incarceration in 9.7% of cases and strangulation in approximately 1% [1].

Inguinoscrotal hernias with loss of domain pose a significant surgical challenge due to the risk of cardiorespiratory complications resulting from the sudden increase in intra-abdominal pressure during the reduction of herniated viscera. This can lead to the development of abdominal compartment syndrome [2]. To prevent these complications, several preoperative strategies have been proposed, including progressive pneumoperitoneum [3] and the use of botulinum toxin [4-6].

Patients with cirrhosis present an increased surgical risk due to potential intraoperative and postoperative complications [7], which are influenced by the degree of hepatic dysfunction, the presence of portal hypertension, the type of surgical procedure, and associated comorbidities [8]. Tools such as the MELD and Child-Pugh scores can help assess the severity of liver disease and guide decision-making regarding elective surgery [9]. Generally, patients classified as Child-Pugh A or with a MELD score below 10 are not considered to have contraindications to elective surgical procedures [10].

## Case presentation

**Figure 1:**
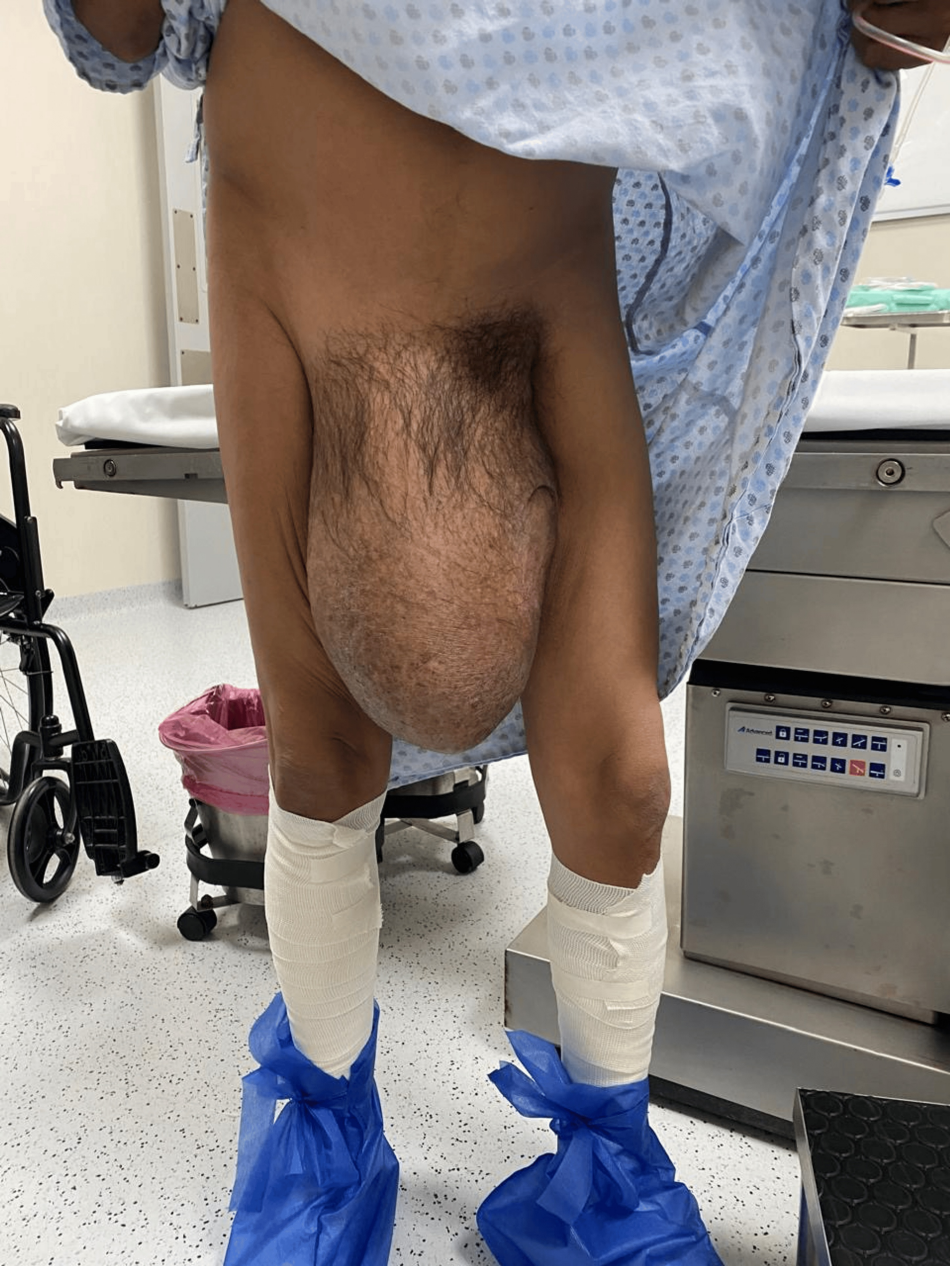
Preoperative view of the inguinoscrotal hernia

**Figure 2:**
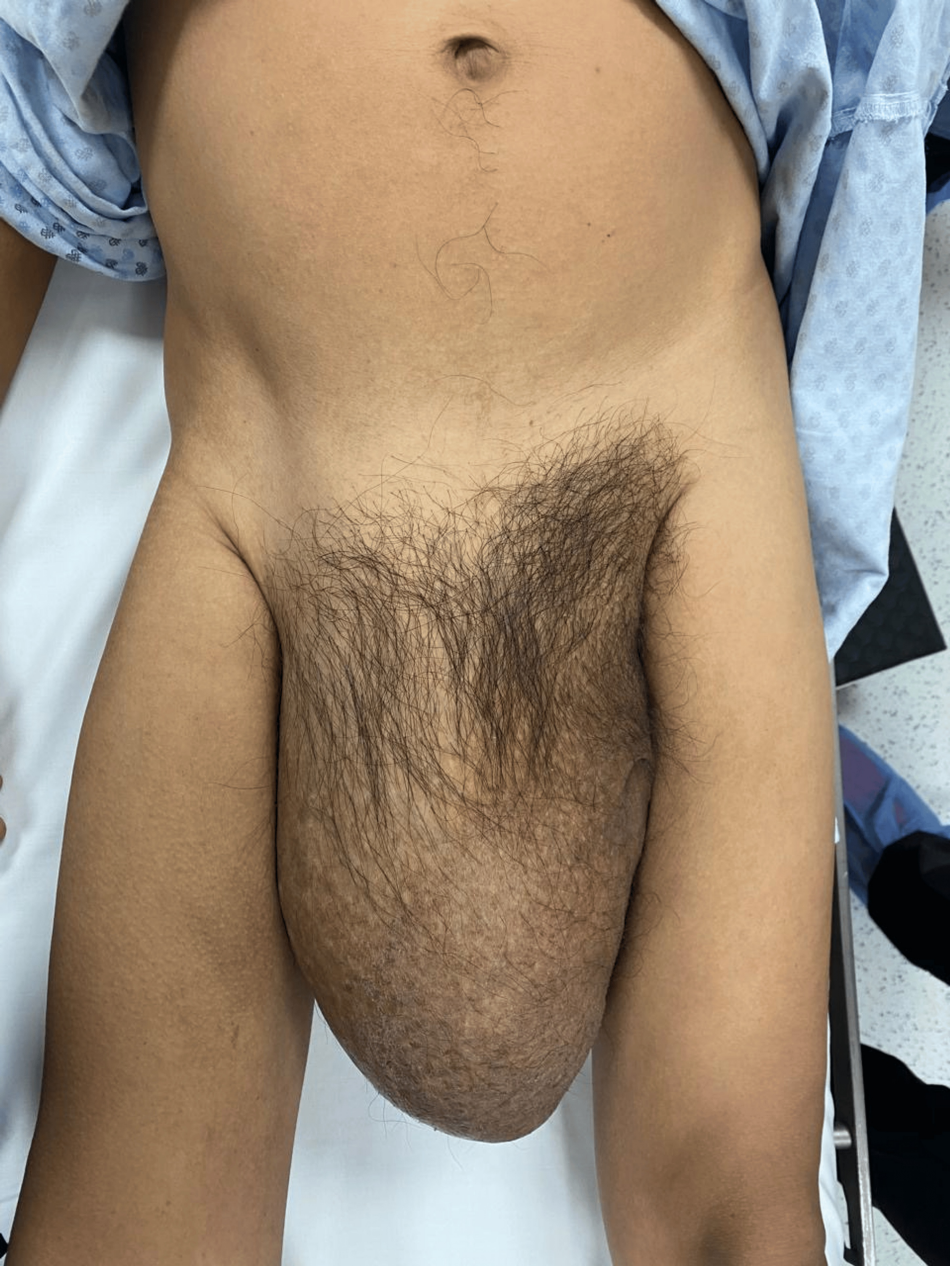
Frontal view of the inguinoscrotal hernia

**Figure 3:**
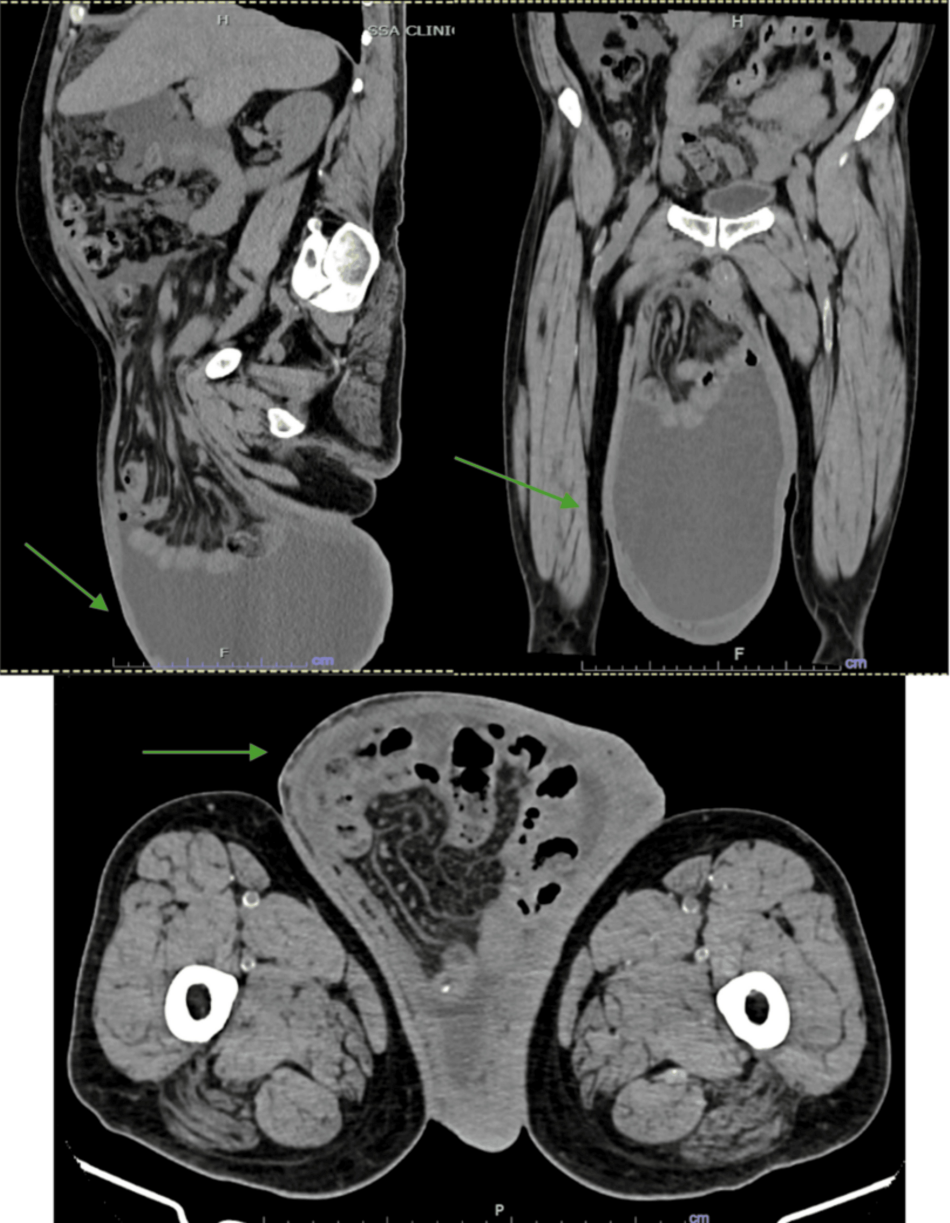
CT scan showing the location of the inguinoscrotal hernia

A 47-year-old male patient with a history of liver cirrhosis, diagnosed six years earlier, presented for consultation after experiencing a progressively enlarging right inguinal hernia for the past 10 years. Physical examination revealed an irreducible inguinoscrotal hernia extending into the distal region of the right thigh, without signs of strangulation (Figure 1, Figure 2). A preoperative CT scan was performed to evaluate the anatomy and calculate the Tanaka index, which was 55% in this case (Figure 3).

**Figure 4:**
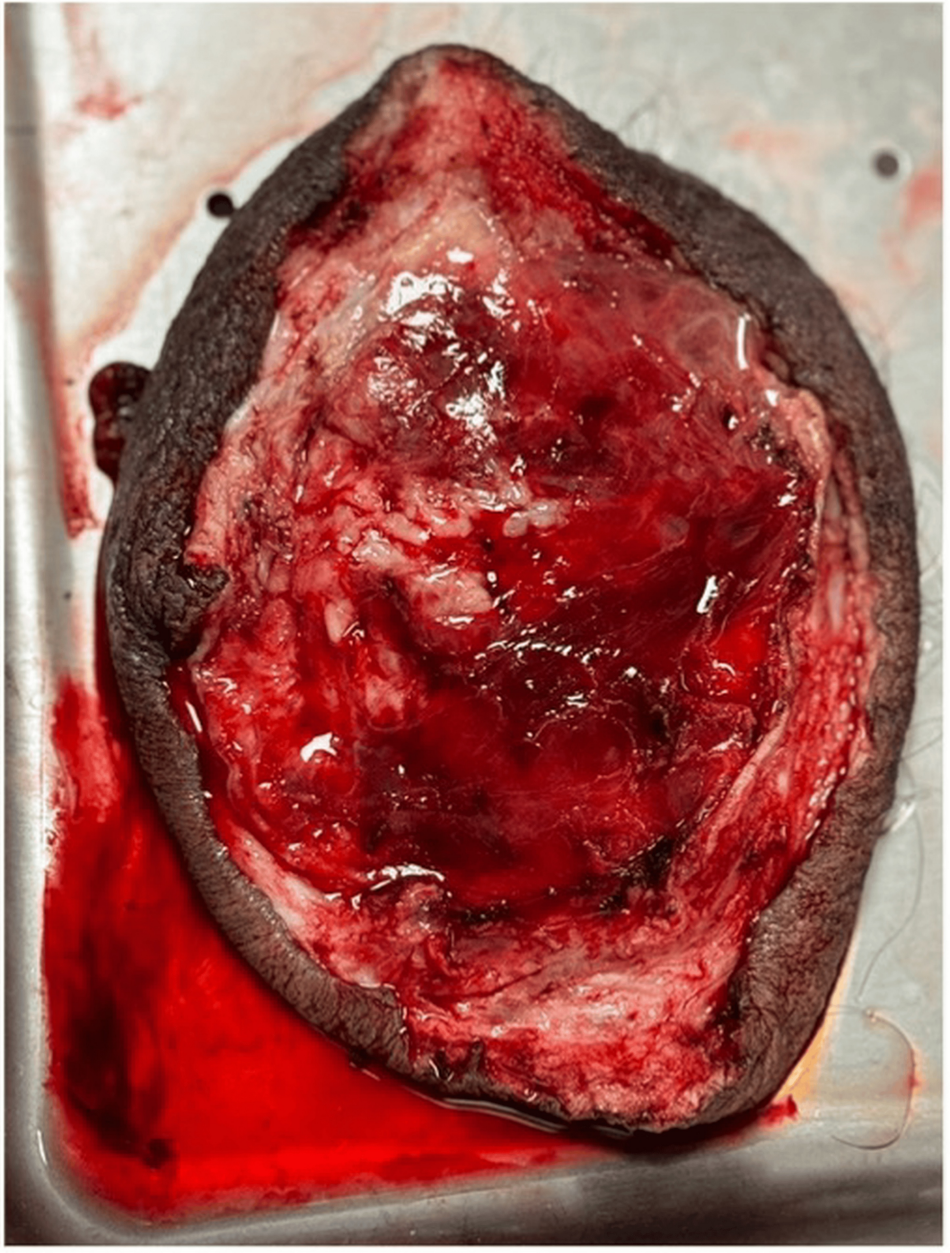
Intraoperative image showing the dissected scrotum

In the operating room, under local anesthesia and sedation, a 7 Fr, 3-way central venous catheter was placed using an open technique, which involved tissue dissection down to the peritoneum and exteriorization through a counter-incision (Figure 4). 

**Figure 5:**
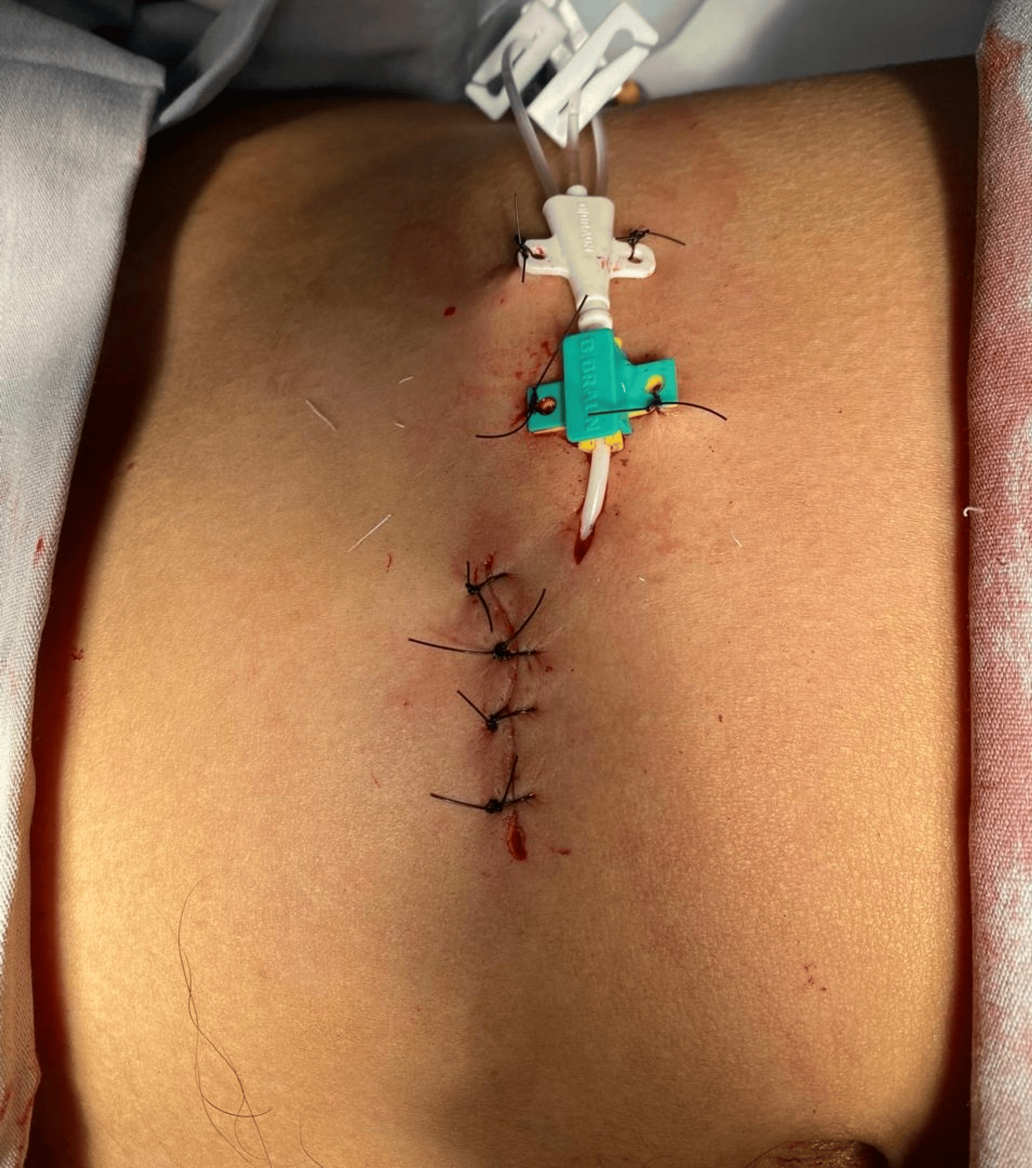
Immediate postoperative result

Six sessions of progressive pneumoperitoneum were carried out to expand the abdominal cavity. Insufflation began with 500 cc of atmospheric air (Figure 5), and the patient was closely monitored for complications such as severe pain or respiratory distress. The volume of insufflated air was gradually increased in each session, reaching 700 cc and, ultimately, 1000 cc. 

Following the final pneumoperitoneum session, 100 IU of botulinum toxin (Nabota) was administered under ultrasound guidance to the oblique muscles of the abdominal wall. The injections were distributed across three points along the anterior axillary line, between the costal margin and the anterior superior iliac spine, resulting in six punctures in total, with approximately 15 IU injected at each site.

An inguinal plasty was performed through a transverse incision, where a hernial sac approximately 2 cm thick was identified and opened. The small bowel loops were dissected and reduced into the abdominal cavity. Intraoperatively, a testicular tumor was discovered, prompting an orchiectomy and subsequent resection of scrotal tissue.

**Figure 6:**
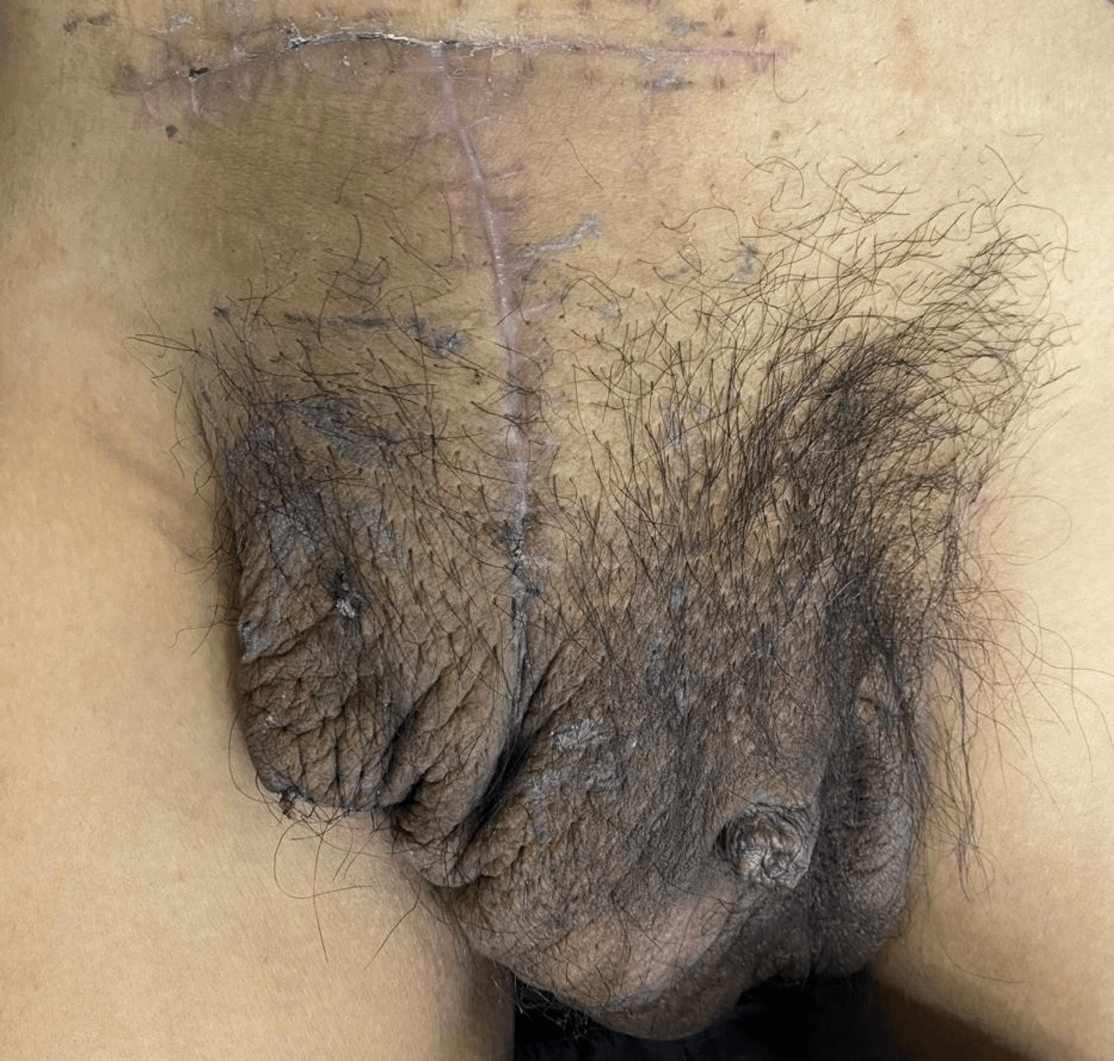
Final outcome during outpatient follow-up

The patient was followed up in the outpatient clinic and showed no postoperative complications (Figure 6). 

## Discussion

The present case stands out for its complexity, as giant inguinoscrotal hernias with loss of domain represent a significant surgical challenge due to the physiological alterations they entail, particularly the risk of abdominal compartment syndrome following abrupt reduction of the herniated contents [1].

These hernias are associated with numerous respiratory and hemodynamic complications, requiring meticulous preoperative planning [2]. One strategy proposed in the literature to facilitate closure and reduce the risk of complications is preoperative progressive pneumoperitoneum. This technique has proven effective by gradually expanding the abdominal cavity, thereby enabling the reintegration of viscera [3].

The presence of liver cirrhosis further complicates the surgical prognosis. These patients are at increased risk of bleeding, infection, and hepatic decompensation, all of which contribute to higher morbidity and mortality [4]. According to Teh et al., cirrhotic patients, particularly those with advanced liver disease, exhibit significantly higher rates of postoperative complications [4].

In this context, the preoperative application of botulinum toxin type A has emerged as a valuable adjunct. By inducing temporary paralysis of the abdominal wall muscles, it enhances abdominal wall compliance and reduces tension during closure [5]. In the present case, botulinum toxin was administered under ultrasound guidance, following established protocols for complex hernia management [5,6].

Tanaka et al. proposed a CT-based method for calculating the ratio between the volume of the hernia sac and the abdominal cavity, an approach useful in predicting the need for additional techniques such as pneumoperitoneum [7]. In this case, the Tanaka index was 55%, fully supporting the preoperative strategy employed.

Furthermore, Kingsnorth and LeBlanc emphasized the need for individualized surgical approaches in giant hernia cases, highlighting the importance of controlling risk factors and selecting the appropriate surgical technique [8]. Schreinemacher et al. also underlined the relevance of proper classification systems for ventral hernias to guide treatment based on defect size and patient condition [9].

Lastly, the Clavien-Dindo classification remains a valuable tool for grading postoperative complications. It provides an objective framework for assessing surgical outcomes and enables more accurate comparison across studies [10].

## Conclusions

The management of giant inguinoscrotal hernias with loss of domain in cirrhotic patients continues to pose a major surgical challenge. A multidisciplinary strategy that includes preoperative optimization techniques, such as progressive pneumoperitoneum and botulinum toxin injection, can significantly mitigate perioperative risks and support successful hernia repair.

This case illustrates that, with careful planning and individualized care, favorable outcomes can be achieved even in high-risk patients with complex abdominal wall defects. Ongoing surgical innovation, grounded in a solid understanding of anatomy and physiology, remains vital in improving care for this patient population. Continued clinical experience and research will be essential to refine these approaches and enhance future prognoses.

## References

[1] Begliardo FL, Arias PM, Corpacci P (2018). Management of giant inguinoscrotal hernia with loss of domain: a surgical challenge [Article in Spanish]. Rev Hispanoam Hernia.

[2] Muñoz HA, González JC (2021). Clinical practice guideline and management of inguinal hernia [Article in Spanish]. Rev Hispanoam Hernia.

[3] Santos-Sánchez O (2018). Surgical risk assessment in patients with liver cirrhosis [Article in Spanish]. Rev Colomb Gastroenterol.

[4] Teh SH, Nagorney DM, Stevens SR (2007). Risk factors for mortality after surgery in patients with cirrhosis. Gastroenterology.

[5] Ibarra-Hurtado TR, Nuño-Guzmán CM, Robles-Campos R, Navarro-Ibarra R, Troyo-Sanroman R (2009). Preoperative botulinum toxin type A injection in complex ventral hernia repair. World J Surg.

[6] Bartolini I, Bechi P (2013). Chylous ascites after laparoscopic anterior resection of the rectum. Surgery.

[7] Tanaka EY, Yoo JH, Rodrigues AJ Jr, Utiyama EM, Birolini D, Rasslan S (2010). A computerized tomography scan method for calculating the hernia sac and abdominal cavity volume in complex large incisional hernia with loss of domain. Hernia.

[8] Kingsnorth AN, LeBlanc KA (2003). Hernias: inguinal and incisional. Lancet.

[9] Schreinemacher MH, van Barneveld KW, Dikmans R, Gijbels MJ, Greve JW, Bouvy ND (2013). Importance of a classification for a tailored surgical approach in ventral hernia repair. Hernia.

[10] Dindo D, Demartines N, Clavien PA (2004). Classification of surgical complications: a new proposal with evaluation in a cohort of 6336 patients and results of a survey. Ann Surg.

